# Prevalence of Dyslipidemia in Students from Han, Uygur, and Kazakh Ethnic Groups in a Medical University in Xinjiang, China

**DOI:** 10.1038/s41598-019-55480-5

**Published:** 2019-12-19

**Authors:** Jialin Abuzhalihan, Yong-Tao Wang, Dilare Adi, Yi-Tong Ma, Zhen-Yan Fu, Yi-Ning Yang, Xiang Ma, Xiao-Mei Li, Fen Liu, Bang-Dang Chen

**Affiliations:** 1 Department of Cardiology, First Affiliated Hospital of Xinjiang Medical University, Urumqi, 830054 P.R. China; 2 Xinjiang Key Laboratory of Cardiovascular Disease Research, Urumqi, 830054 P.R. China

**Keywords:** Dyslipidaemias, Interventional cardiology

## Abstract

In the present study, we aimed to evaluate the prevalence of dyslipidemia in students from different ethnic groups in Xinjiang. It is an observational, cross-sectional study. The sample of 7096 students aged 21–25 years was randomly selected from the clinic of Xinjiang Medical University. Baseline data, serum concentration of total cholesterol (TC), triglyceride (TG), low density lipoprotein cholesterol (LDL-C), high density lipoprotein cholesterol (HDL-C) and fasting plasma glucose (FPG) were reported. The prevalence of changes in lipid profile according to Body mass index (BMI) in three ethnic groups was calculated. Compared with Han and Uygur students, TC, LDL-C, TG and FPG levels were lower in kazakh sutdents, while HDL-C level was lower in Uygur students. The prevalence of high TC change was higher in Uygur students, and high LDL-C change was higher in Han students. The prevalence of low HDL-C change was higher in Uygur students, and high TG change was lower in Kazakh students. The prevalence of high TC, LDL-C, TG and low HDL-C changes was observed in normal weight, overweight and obesity groups according to the nutritional status by BMI among students of each ethnic group. The present study demonstrated the prevalence of dyslipidemia in students from different ethnic groups, and enriched the limited data on the early prevention and treatment of dyslipidemia and cardiovascular diseases in Xinjiang medical students crowd.

## Introduction

Atherosclerosis is as a result of pathological accumulation of plaque in the arterial vessels and leads to cardiovascular disease, the main cause of death in the worldwide^1^. It is well known that atherosclerotic process begins early in childhood and dyslipidemia plays a vital role in the progression of the disease^2^. Dyslipidemia is a family of lipoprotein metabolism disorders manifested by elevated total cholesterol (TC), low-density lipoprotein cholesterol (LDL-C), triglycerides (TG), and reduced high density lipoprotein cholesterol (HDL-C) concentrations in the blood^3^. Increased lipid levels result in vessel wall reations, including endothelial dysfunction, smooth muscle cells proliferation, lipid accumulation, foam cell formation, and, finally, necrosis and plaque development^4,5^. Like other well-known risk factors such as diabetes, hypertention, obesity and smoking, dyslipidemia is also associated with the development of atherosclerotic disease^6–8^. Studies revealed that early onset of dyslipidemia is associated with the development of early atherosclerotic coronary and peripheral artery disease and increased incidence of cardiovascular disease in adulthood^9,10^.

Recently, dyslipidemia is increasingly prevalent in all age groups, and the incidence tends to be younger^11^. In the last decade, Diseases caused by high total cholesterol (TC) increased global morbidity and mortality by 26.9% and 28.0%, respectively^12,13^. Bogalusa Heart Study reported that more than 70% of children with adverse lipid profiles are prone to dyslipidemia in adult life^14^. A study conducted on individuals from two regions of Argentina, aged 7–14 years old, revealed the prevalence of high TG was 28.8% and 3.5% and low HDL was 30% and 5.5%^15^. Another study on the Korean students aged 10–18 years old, indicated that the morbidity of dyslipidemia in girls and boys was 21.7% and 25.2%, respectively^16^. Greek school-aged boys were found to have higher mean of TC (5.8 mg/dL) and TG (10.8 mg/dL), and lower mean of HDL-C (−16.9 mg/dL)^17^. Identification and prevention of dyslipidemia at the young age is an important strategy to reduce present and future health risks.

Epidemiologic surveys of cardiovascular disease (CVD) precursors in children have showed that differences in lipid and lipoprotein levels among cultures and ethnic groups occur early in childhood^18,19^. Further, we assumed that college students are not as disrupted by environmental factors as adults, and genetic factors may be considered as the main influencing factors of dyslipidemia. In the present study, we aimed to establish the prevalence of dyslipidemia in college students from different ethnic groups and to evaluate its association with the nutritional status.

## Methods

This is an observational, cross-sectional study, and the sample was obtained from students who were seen at the clinic of the Xinjiang Medical University from September 2018 to December 2018, consisting of Han, Uygur, and Kazakh students aged between 21 and 25, of both genders. Trained research interviewers administered standardized questionnaire through in-person interviews. Data collection involved demographic information, dietary habit, physical activity, and medical historty. Anthropometric measures (e.g. height, weight, and blood pressure) were performed according to a standard protocol. Blood samples were obtained from an antecubital vein into vacutainer tubes containing EDTA in the morning after an overnight fasting period. All the collected samples were transported on dry ice at prearranged intervals to Xinjiang coronary artery disease VIP laboratory. The concentration of serum total cholesterol, triglyceride, low density lipoprotein (LDL), high density lipoprotein (HDL) and fasting glucose were measured by the Clinical Laboratory Department of the First Affiliated Hospital of Xinjiang Medical University with the biochemical analyzer (Dimension AR/AVL Clinical Chemistry System, Newark, NJ, USA). Each participant was informed and signed a written consent. The survey protocol abided the ethical guidelines of the 1975 declaration of Helsinki^20,21^.

The 2016 Chinese Guideline for the Management of Dyslipidemia in Adults (Chinese guideline) was used to classify the serum TC, LDL-C, HDL-C, and TG levels^22^. These classifications defined by Chinese guideline were the same with the criteria in the Third Report of the National Cholesterol Education Program (NCEP) Expert Panel on Detection, Evaluation, and Treatment of High Blood Cholesterol in Adults (Adult Treatment Panel III) final report (NCEP-ATP III)^23^. High TC was defined as TC ≥ 6.22 mmol/L. High LDL-C was defined as LDL-C ≥ 4.14 mmol/L. Low HDL-C was defined as HDL-C ≤ 1.04 mmol/L, and high TG was defined as TG ≥ 2.26 mmol/L. Body mass index (BMI, kg/m^2^) was calculated from the height and weight measurements. Based on the Criteria of Weight for Adults released by the Ministry of Health of China (WS/T 428–2013), individuals were categorized into three groups: 18.5 kg/m^2^ ≤BMI< 24.0 kg/m^2^ (normal weight), 24.0 kg/m^2^ ≤BMI < 28.0 kg/m^2^ (over weight), and BMI ≥ 28.0 kg/m^2^ (obesity)^21^.

Exclusion criteria included (1) no blood samples available; (2) missing data on lipid concentrations; (3) ethnic groups other than Han, Uygur and Kazakh; (4) known familial hypercholesterolemia; (5) use of cholesterol-lowering medications; (6) age younger than 21 years and older than 25 years. A total of 7096 students were finally enrolled in the present study.

### Statistical analysis

The analysis was performed using SPSS 22 software. The continuous variables were presented as mean and standard deviation and the categorical variables as absolute frequency. The distribution of variables was assessed using the Kolgomorov-Sminorv test. The comparisons of the continuous variables with normal distribution were performed with the unpaired Student’s t-test, and for more than two independent groups, one way variance analysis (ANOVA) was used. Following the the ANOVA with statistically significant results, post hoc Tukey comparisons were performed, and all p-values were adjusted. For the comparisons of categorical variables, the chi-square test or Fisher’s exact test was used. For the study of the association, multiple linear regression were performed. P value < 0.05 was considered statistically significant.

### Ethics approval and consent to participate

The study was approved by the Ethical Review Board of The First Affiliated Hospital of Xinjiang Medical University. Written informed consent was obtained from all enrolled patients.

## Results

Baseline characteristics and the mean values of lipid profile of the population enrolled according to ethnic groups were listed in Table 1. A total of 7096 students from three ethnic groups with a mean age of 21.26 ± 1.64 years were participated in this project, and 2451(34.5%) of them were male. Compared with Han and Kazakh students, the average height and weight of Uygur students were lower. BMI values were higher in Han students than other two groups, while blood pressure was lower in Uygur students than others. TC, LDL-C, TG and FPG levels were lower in Kazakh students than other two groups, and HDL-C levels were lower in Uygur students than Han and Kazakh students (all P < 0.05, Table 1).

**Table 1:** Characteristics of study subjects.

Variable	No.(%) or Mean ± SD				P value
	Total (7096)	Han (3426)	Uygur (3007)	Kazakh (663)	
Age	21.26 ± 1.64	20.87 ± 1.51**	21.63 ± 1.69*	21.59 ± 1.59*	<*0*.*001*
Male(%)	2451 (34.5)	1328 (38.8)**	936 (31.1)*	187 (28.2)*	<*0*.*001*
Weight(Kg)	59.99 ± 11.90	60.88 ± 12.7*	58.88 ± 11.01**	60.36 ± 11.11*	<*0*.*001*
Height(cm)	166.97 ± 8.81	167.63 ± 8.61*	166.10 ± 9.16**	167.45 ± 7.82*	<*0*.*001*
BMI(Kg/m^2^)	21.39 ± 3.14	21.53 ± 3.35*	21.23 ± 2.90*	21.39 ± 2.99	*0*.*001*
SBP(mmHg)	110.39 ± 13.24	111.28 ± 13.33*	109.18 ± 12.88**	111.31 ± 13.95*	<*0*.*001*
DBP(mmHg)	69.75 ± 7.42	70.45 ± 9.58*	68.83 ± 9.02**	70.38 ± 9.94*	<*0*.*001*
TC(mmol/L)	3.96 ± 0.68	3.97 ± 0.68*	3.95 ± 0.67*	3.87 ± 0.70**	*0*.*001*
HDL-C(mmol/L)	1.43 ± 0.30	1.44 ± 0.30*	1.40 ± 0.29**	1.44 ± 0.31*	<*0*.*001*
LDL-C(mmol/L)	2.19 ± 0.58	2.19 ± 0.60*	2.20 ± 0.57*	2.10 ± 0.59**	<*0*.*001*
TG(mmol/L)	0.87 ± 0.48	0.93 ± 0.53**	0.82 ± 0.44*	0.76 ± 0.35*	<*0*.*001*
FPG(mmol/L)	4.80 ± 0.45	4.87 ± 0.47**	4.75 ± 0.42*	4.72 ± 0.40*	<*0*.*001*

Statistically significant values are in italics.

BMI: body mass index; SBP: systolic blood pressure; DBP: diastolic blood pressure; TC: total cholesterol; HDL-C: high density lipoprotein- cholesterol; LDL-C: low density lipoprotein-cholesterol; TG: Triglyceride; FPG: fasting plasma glucose.

All p values are adjusted.

*Compared with only one group P < 0.05; **Compared with the other two groups P < 0.05.

Regarding the prevalence of lipid profile changes, according to different ethnic groups, it was observed the prevalence of high TC change was higher in Uygur students, but the difference was not statistically significant (P = 0.8, Table 2). The prevalence of high LDL-C change was significantly higher in Han students (P = 0.003, Table 2), and the prevalence of low HDL-C change was significantly higher in Uygur students (P < 0.001, Table 2). Meanwhile, the prevalence of high TG change was significantly lower in Kazakh students (P < 0.001, Table 2).

**Table 2:** Prevalence of dyslipidemia according to ethnic groups.

Lipids	Total	Han	Uygur	Kazakh	P value
**TC** (**mg/dl)**					
Normal	7005 (98.7%)	3383 (98.7%)	2966 (98.6%)	656 (98.9%)	0.800
Changed	91 (1.3%)	43 (1.3%)	41 (1.4%)	7 (1.1%)	
**LDL-C** (**mg/dl)**					
Normal	6620 (93.3%)	3179 (92.8%)	2802 (93.2%)	639 (96.4%)	*0*.*003*
Changed	476 (6.7%)	247 (7.2%)	205 (6.8%)	24 (3.6%)	
**HDL-C** (**mg/dl)**					
Normal	5769 (81.3%)	2841 (82.9%)	2373 (78.9%)	555 (83.7%)	<*0*.*001*
Changed	1327 (18.7%)	585 (17.1%)	634 (21.1%)	108 (16.3%)	
**TG** (**mg/dl)**					
Normal	6737 (94.9%)	3199 (93.4%)	2888 (96.1%)	650 (98.1%)	<*0*.*001*
Changed	359 (5.1%)	227 (6.6%)	119 (3.9%)	13 (1.9%)	

Statistically significant values are in italics.

The prevalence of changes in lipid profile according to the nutritional status by BMI in three ethnic groups was listed in Tables 3–5. In Han students, It was observed that the prevalence of high TC, LDL-C and low HDL-C change was higher in obese students, while prevalence of high TG change was higher in normal weight students, but the differences were not statistically significant. (Table 3). In Uygur students, the higher prevalence of high TC, LDL-C and low HDL-C change was observed in overweight students, and high TG change in normal weight students, but the differences were not statistically significant (Table 4). However, in Kazakh students, the higher prevalence of low HDL-C change was mainly concentrated in obese students (P < 0.001, Table 5). The prevalence of high TC change was higher in normal weight students, while the prevalence of high LDL-C and high TG change were higher in obese students, but there were also not statistically significant differences (Table 5).

**Table 3:** Prevalence of dyslipidemia according to nutritional status by BMI in Han students.

Lipids	Nutritional diagnosis				P value
	Total (3240)	Normal weight (2265)	Overweight (517)	Obesity (458)	
**TC(mg/dl)**					
Normal	3203 (98.9%)	2241 (98.9%)	511 (98.8%)	451 (98.5%)	0.69
Changed	37 (1.1%)	24 (1.1%)	6 (1.2%)	7 (1.5%)	
**LDL-C** (**mg/dl)**					
Normal	3019 (93.2%)	2116 (93.4%)	479 (92.6%)	424 (92.6%)	0.705
Changed	221 (6.8%)	149 (6.6%)	38 (7.4%)	34 (7.4%)	
**HDL-C** (**mg/dl)**					
Normal	2694 (83.1%)	1882 (83.1%)	436 (84.3%)	376 (82.1%)	0.642
Changed	546 (16.9%)	383 (16.9%)	81 (15.7%)	82 (17.9%)	
**TG** (**mg/dl)**					
Normal	3030 (93.5%)	2109 (93.1%)	487 (94.2%)	434 (94.8%)	0.337
Changed	210 (6.5%)	156 (6.9%)	30 (5.8%)	24 (5.2%)	

Statistically significant values are in italics.

**Table 4:** Prevalence of dyslipidemia according to nutritional status by BMI in Uygur students.

Lipids	Nutritional diagnosis				P value
	Total (2923)	Normal weight (2104)	Overweight (450)	Obesity (369)	
**TC (mg/dl)**					
Normal	2886 (98.7%)	2080 (98.9%)	441 (98.0%)	365 (98.9%)	0.316
Changed	37 (1.3%)	24 (1.1%)	9 (2.0%)	4 (1.1%)	
**LDL-C** (**mg/dl)**					
Normal	2725 (93.2%)	1967 (93.5%)	413 (91.8%)	345 (93.5%)	0.413
Changed	198 (6.8%)	137 (6.5%)	37 (8.2%)	24 (6.5%)	
**HDL-C** (**mg/dl)**					
Normal	2303 (78.8%)	1660 (78.9%)	348 (77.3%)	295 (79.9%)	0.644
Changed	620 (21.1%)	444 (21.1%)	102 (22.7%)	74 (20.1%)	
**TG** (**mg/dl)**					
Normal	2807 (96.0%)	2013 (95.7%)	438 (97.3%)	356 (96.5%)	0.235
Changed	116 (4.0%)	91 (4.3%)	12 (2.7%)	13 (3.5%)	

Statistically significant values are in italics.

**Table 5:** Prevalence of dyslipidemia according to nutritional status by BMI in Kazakh students.

Lipids	Nutritional diagnosis				P value
	Total (641)	Normal weight (469)	Overweight (80)	Obesity (92)	
**TC(mg/dl)**					
Normal	634 (98.9%)	463 (98.7%)	80 (100%)	91 (98.9%)	0.596
Changed	7 (1.1%)	6 (1.3%)	0 (0%)	1 (1.1%)	
**LDL-C** (**mg/dl)**					
Normal	615 (95.9%)	452 (96.4%)	77 (96.3%)	86 (93.5%)	0.432
Changed	26 (4.1%)	17 (3.6%)	3 (3.7%)	6 (6.5%)	
**HDL-C** (**mg/dl)**					
Normal	548 (85.5%)	399 (85.1%)	78 (97.5%)	71 (77.2%)	*0*.*001*
Changed	93 (14.5%)	70 (14.9%)	2 (2.5%)	21 (22.8%)	
**TG** (**mg/dl)**					
Normal	629 (98.1%)	459 (97.9%)	80 (100%)	90 (97.8%)	0.418
Changed	12 (1.9%)	10 (2.1%)	0 (0%)	2 (2.2%)	

Statistically significant values are in italics.

For all the varabiles, multiple linear regression analysis, according to different ethnic groups, was performed to estimate the risk factors of BMI. The results revealed that, in Han students (R^2^ = 0.310), Age (β = −0.036, P = 0.013), Sex (β = −0.066, P < 0.001), HDL-C (β = −0.242, P < 0.001), LDL-C (β = 0.174, P = 0.018), TG (β = 0.138, P < 0.001), FPG (β = 0.031, P = 0.038), SBP (β = 0.127, P < 0.001) and DBP (β = 0.098, P < 0.001) were the independent risk factors of BMI (Table 6). In Uygur students (R^2^ = 0.269), Sex (β = −0.092, P < 0.001), TC (β = −0.328, P = 0.004), LDL-C (β = 0.466, P < 0.001), TG (β = 0.159, P < 0.001), SBP (β = 0.099, P < 0.001) and DBP (β = 0.119, P < 0.001) were the independent risk factors of BMI (Table 7). In Kazakh students (R^2^ = 0.230), only Sex (β = −0.145, P < 0.001), TG (β = 0.117, P = 0.012) and DBP (β = 0.148, P = 0.011) were the independent risk factors of BMI (Table 8).

**Table 6:** Multivariate linear regression analysis between baseline characteristics and BMI in Han students.

Variable	Regression coeffcient	95%CI	P value
Age	−0.036	−0.099~0.027	*0*.*013*
Sex	−0.066	−0.291~0.159	<*0*.*001*
TC (mmol/L)	−0.011	−0.742~0.720	0.89
HDL-C (mmol/L)	−0.242	−1.104~0.620	<*0*.*001*
LDL-C (mmol/L)	0.174	−0.632~0.980	*0*.*018*
TG (mmol/L)	0.138	−0.109~0.385	<*0*.*001*
FPG (mmol/L)	0.031	−0.177~0.239	*0*.*038*
SBP (mmHg)	0.127	0.008~0.245	<*0*.*001*
DBP (mmHg)	0.098	0.082~0.114	<*0*.*001*

Statistically significant values are in italics.

**Table 7:** Multivariate linear regression analysis between baseline characteristics and BMI in Uygur students.

Variable	Regression coeffcient	95%CI	P value
Age	0.010	−0.048~0.068	0.524
Sex	−0.092	−0.325~−0.141	<*0*.*001*
TC (mmol/L)	−0.328	−0.290~−0.634	*0*.*004*
HDL-C (mmol/L)	−0.048	−1.146~1.050	0.394
LDL-C (mmol/L)	0.466	−0.596~1.528	<*0*.*001*
TG (mmol/L)	0.159	−0.143~0.461	<*0*.*001*
FPG (mmol/L)	0.020	−0.205~0.245	0.232
SBP (mmHg)	0.099	−0.019~0.217	<*0*.*001*
DBP (mmHg)	0.119	0.103~0.135	<*0*.*001*

Statistically significant values are in italics.

**Table 8:** Multivariate linear regression analysis between baseline characteristics and BMI in Kazakh students.

Variable	Regression coeffcient	95%CI	P value
Age	−0.004	−0.135~0.127	0.912
Sex	−0.145	−0.674~0.384	<*0*.*001*
TC (mmol/L)	−0.136	−2.617~2.345	0.65
HDL-C (mmol/L)	−0.122	−2.823~2.579	0.394
LDL-C (mmol/L)	0.264	−2.502~3.030	0.34
TG (mmol/L)	0.117	−0.665~0.899	*0*.*012*
FPG (mmol/L)	0.019	−0.510~0.548	0.607
SBP (mmHg)	0.029	0.005~0.053	0.616
DBP (mmHg)	0.148	0.113~0.183	*0*.*011*

Statistically significant values are in italics.

## Discussion

It is well known that lipid disorders in children and adolescents are associated with increased risks of cardiovascular and cerebrovascular disease in adults^24^. Hence, for improving cardiovascular health in adulthood, dyslipidemia in children and adolescents should be screened early^25^. Studies reported that lipid metabolism disorders, such as high LDL-C and low HDL-C, in children could be used as the main predictors of future atherosclerosis^26^. A study on Spanish children showed that the reduced mortality from ischemic cardiac disease in children may be associated with elevated HDL-C levels compared with other developed countries^27^. Therefore, dyslipidemia in adolescents or children should be taken attention.

Previous studies on lipidomics reported that there are differences between races in lipid levels. The Bogalusa Heart Study reported that the black children had higher mean levels of TC, LDL-C and HDL-C than white children did^28^. The NHANES III data showed that, compared with non-Hispanic white and Mexican Americans’, the TC, LDL-C, and HDL-C levels were higher in non-Hispanic black children and adolescents^29,30^. Xinjiang locates in the northwest of China, where the main ethnic groups are Han, Uygur and Kazakh. Since the geographical location, living environment and ethnic categories of Xinjiang province are different from other parts of China, the life styles of the people are also quite different. As the most Kazakh people lead nomadic lives, the consumption of beef, mutton and dairy products is much higher than others. Uygur people are mainly based on agriculture, the levels of pasta and meat consumption are higher than Han people. Furthermore, Xinjiang is defined as the area of high incidence of cardiovascular diseases in China^31^.

In present study, we analyzed serum concentrations of TC, LDL-C, HDL-C, and TG in students from Han, Uygur and Kazakh ethnic groups, and evaluated the association of overweight and obesity with these variables. Our study showed that although there were significant differences in LDL-C, HDL-C and TG among the three groups, the difference was not significant in TC. For LDL-C levels, Kazakh students had obviously lower values than other two groups, while Uygur students had apparently high HDL-C lowering values. Han students had the highest values of TG among the three groups. Besides of the ethnic factor, the main reason of these differences may be life style, Uygurs and kazakhs consume more pasta, meat and dairy products than Han do, which are higher in saturated and trans fats. This may suggest that, in Xinjiang, the prevalence of dyslipidemia among these ethnic groups has been different since adolescence even childhood. Nevertheless, We have found that although the differences in mean values between the three groups are small, the statistical analysis is still significant even after adjustment for multiple comparisons. This is probably an effect of the large sample size.

Obesity is not only a disease itself, but also a risk factor for other diseases. It is an increasingly serious global problem^32^. In the Europe, 30–40% of children between the ages of 6 and 10 are overweight (including overweight and obesity)^24^, and up to 43% of American children and adolescents with overweight/obesity diagnosed as dyslipidemia^25^. Similarly, obesity persists into adulthood, and is positively correlated with a lot of diseases, including hypertension, diabetes, dyslipidemia, insulin resistance (IR), and atherosclerosis^33,34^. Obesity rates are still rising rapidly in many parts of the world, and if current trends continue, they will reach 18% of men and more than 21% of women globally by 2025, placing a heavy burden on individuals, societies and health care systems^35^. Consequently, unless prevented or treated promptly, this risk factor is maintained throughout early adulthood, suggesting the need for early preventive intervention.

When analyzing lipid profile of each ethnic group students according to the nutritional status by BMI, in Han students, the prevalence of high TC, LDL-C, low HDL-C was concentrated in the obese group and high TG in the normal weight group. In Uygur students, the overweight group had the higher prevalence of high TC, LDL-C, and low HDL-C than other groups did, while the normal weight group had the highest prevalence of high TG. In Kazakh students, the prevalence of high LDL-C, TG and low HDL-C was observed in the obese group, and high TC was in the normal weight group. This may indicate a high incidence of dyslipidemia in Kazakh populations, even if they are not overweight or obese. Besides of the ethnic difference and life styles, genetic factors that mediate the levels of lipid proteins may play a role in the synthesis of serum lipid.

The main strength of the study was a large cohort of healthy students from different ethnic groups. We compared the blood lipid levels of medical students from three different ethnic groups and laid the foundation for preliminarily evaluating the distribution of abnormal blood lipid in these ethnic groups. In addition, the cohort of overweight/obese students was collected during the same period, which provided information on the effect of obesity on lipid concentration, thus clarifying the health-related effects of obesity on human metabolism in early childhood. Therefore, our results support the development of early prevention strategies for cardiovascular disease in this region. The limitation of this study is its observational, cross-sectional sample population, and our sample is from students at Xinjiang Medical University. Although these students come from all parts of Xinjiang, they could not represent the young population of Xinjiang. Further researches could utilize the results suggested in this article to study the associated risk factors and intervention of overweight and obesity in a representative sample of the adult population.

In conclusion, this study demonstrated the prevalence of dyslipidemia in different ethnic group students. The continuous relation between cardiovascular disease risk factors and dyslipidemia were documented. This study enriched the limited data on the early prevention and treatment of dyslipidemia and cardiovascular diseases in Xinjiang medical students crowd.

## Data Availability

The data will not be shared, since part of the data is being reused by another study.
